# Does higher health literacy lead to higher trust in public hospitals?

**DOI:** 10.1186/s12939-021-01528-w

**Published:** 2021-09-16

**Authors:** Maja Bertram, Urs Steiner Brandt, Rikke Klitten Hansen, Gert Tinggaard Svendsen

**Affiliations:** 1 Department of Public Health, Unit for Health Promotion Research, University of Southern Denmark, Esbjerg, Denmark; 2 Department of Sociology, Environmental and Business Economic, University of Southern Denmark, Esbjerg, Denmark; 3 Department for Planning and Finances, Odense University Hospital, Odense, Denmark; 4 Department of Political Science, Aarhus University, Aarhus, Denmark

**Keywords:** Health literacy, Social groups, Equity, Trust, Public hospitals, Game theory, Patient satisfaction

## Abstract

**Background:**

Does higher health literacy lead to higher trust in public hospitals? Existing literature suggests that this is the case since a positive association between the level of health literacy and the level of trust in physicians and the health care system has been shown. This study aims to challenge this assumption.

**Methods:**

Based on theoretical arguments from game theory and analysis of empirical data, we argue that the association is better described as an inversely u-shaped curve, suggesting that low and high levels of health literacy lead to a lower level of trust than a medium level of health literacy does. The empirical analysis is based on a study of the Danes’ relationship to the overall health care system. More than 6000 Danes have been asked about their overall expectations of the health service, their concrete experiences and their attitudes to a number of change initiatives.

**Results:**

Game theory analysis show that the combined perceived cooperation and benefit effects can explain an inversely u-shaped relationship between social groups and trust in the health care system. Based on quantitative, binary regression analyses of empirical data, the lowest degree of trust is found among patients from the lowest and highest social groups, while the highest degree of trust is found in the middle group. The main driver for this result is that while patients having low health literacy perceive that the health care system is not cooperative, patients with a high level of health literacy have high expectations about the quality, which the health care system might not be able to provide. This reduces the perceived benefit from their encounter with the health care system.

**Conclusion:**

It is important that health care professionals understand that some patient groups have a higher chance of cooperation (e.g., agreeing on the choice of treatment) or defection (e.g. passing a complaint) than others. In perspective, future research should undertake further qualitative examinations of possible patient types and their demands in relation to different health care sectors, focusing specifically on the opportunities to improve the handling of different patient types.

## Introduction

Effective utilisation of resources and a well-functioning health care system that is both efficient and provides high-quality services is important in order to maintain the welfare-funded health care system [1]. Thus, for society, the delivery of high quality is important, and this is equally important for the individual patient. A 2015 survey by the Centre for Patient Experience and Evaluation examined various patient groups to find out what is important in the encounter with the health care system. Here, a high level of trust in the health care system was crucial for most patients [2]. Several other studies show that trust is the very foundation for a good and well-functioning health care system [3, 4].

Many factors in the encounter between health care system and patient are affected by the degree of trust shown by the patient. In the health care system, a low degree of trust means an increased probability of the patient looking for another physician (second opinion), omitting to use the health care system, poor patient experience, delayed consultations, suppression of necessary patient information, taking out private health insurances and poor cooperation. On the other hand, a high degree of trust is connected to high compliance, patient satisfaction, responsiveness to treatment, continuity in relation to practitioner and use of the health care system whenever needed [5, 6]. The influence exerted by the degree of trust has been highlighted in several studies that have singled out trust as the most important factor for successful treatment [3, 7].

Thus, trust is paramount for the individual’s experience of the health care system, but it is also of great importance to society. Trust in the health care system has generally been high but has begun to decline, and this is one of the reasons why recent years have witnessed an increasing interest in researching trust in the health care system [4, 8, 9].

A Danish survey shows that less than half of the Danes express great trust in the fact that they will receive the right treatment if they become ill. One fourth fear that they will not receive the medicine they need, and one third fears receiving medicine they do not need. More than half of the population believe that it may be necessary to insist in order to receive proper examinations and treatments [10]. Overall, the survey indicates that there may be reason for concern and that it would be advisable to focus further on the problems related to trust in the public health care system.

A relevant approach to investigating the problems further is to gain insight into the possible correlation between trust and social group. Several studies show that the use of the health care system differs depending on the patient’s social group [11]. Levinsen argues that in spite of the relatively high degree of trust in Denmark, this does not mean that there are no social and cultural differences or areas in society where trust is absent or threatened by segregation [12]. Several other studies likewise argue that it may be interesting to study social groups as the source of variations in trust [9, 13]. However, this correlation has not yet been studied [3, 4, 14].

Earlier studies have focused on mapping of trust and comparison between countries [15–17], and studies that have examined variations within one country have primarily focused on race [6, 18, 19]. In addition to this, there is a growing interest in understanding the role of health literacy in relation to trust in the health care system. Studies have shown that higher levels of health literacy led to higher levels of trust in physicians and in the health care system [20–22]. We are curious to investigate whether the association between health literacy and trust can be more complex than this. Thus, we aim to challenge this finding based on the idea that higher social groups with a high level of health literacy might have high demands and a risk of being disappointed. Thus, our main research question is the following:

*Does higher health literacy lead to higher trust in public hospitals?*

In the following, this research question is answered by a mixed method approach with both qualitative and quantitative elements. We begin with a theory section that defines trust, health literacy and their relationship. Based on this theoretical framework, we carry out the formal modelling of the interaction between patients and health personnel followed by a method section and the empirical results. Finally, a discussion and conclusion are given.

### Theory

#### Trust

There are many different definitions of trust, but a common denominator for most definitions is that trust is about positive expectations. Rowe and Calnan believe that trust relations are characterised by the trust giver having positive expectations of the competences of the trust taker and that the trust taker is willing to work in the best interest of the trust giver [9]. Trust can be defined as “an expectation that 1) a person you know, 2) a stranger or 3) a formal institution will adhere to a given norm” [23].

In other words, trust implies the expectation that another person or an employee in a formal institution will not break the standard and cheat you even if there may be some private gain connected to doing so [24]. According to Rowe and Calnan, in order to create trust, it is also important that the trust taker has the competences necessary to perform the task [9].

Trust may be classified in many ways, but in the literature, the predominant classification divides trust into three different types: specific trust, social trust and institutional trust. Specific trust is trust in someone you already know and originates from past experiences with specific people. Social trust is trust in a stranger whom you did not meet previously [25].

While the first two forms of trust are about trusting a person, institutional trust is about trusting an institution, that is, whether citizens have trust in the judicial system, the police, the health care system, the tax authorities and so on. In the following, we focus on institutional trust in public hospitals by looking at health literacy followed by game theory.

#### Health literacy

##### Definition of health literacy

Health literacy can be defined in many ways. One of the most widespread is Nutbeam’s definition:

The cognitive and social skills which determine the motivation and ability of individuals to gain access to, understand, and use information in ways which promote and maintain good health. (It) means more than being able to read pamphlets and make appointments. By improving peoples’ access to health information and their capacity to use it effectively, health literacy is critical to empowerment [26].

Health literacy is thus about having the ability to acquire and understand knowledge, but also to be able to use the acquired knowledge. Health literacy is a mixture of cognitive and social competences that, combined, provide the individual with the ability to access, understand, use and act on the acquired health information [27].

Health literacy is categorised into three different levels, depending on the degree of fulfilment of the above definition. The three different levels are functional health literacy, interactive health literacy and critical health literacy. These different levels reflect an increasingly large autonomy and personal empowerment in decision making as well as commitment and knowledge about health [27]. The various health literacy levels are elaborated in the next sections.

##### Functional health literacy

Functional health literacy concerns the ability to have sufficient reading and writing skills to help you understand and act on health-related information [26]. This could be reading and understanding a patient information pamphlet and, on that basis, being able to take the correct dose of medicine.

A study of 29,000 Danes showed that approximately one out of five Danes experiences problems understanding health information and engaging in a dialogue with the HPs [28]. Hence, having a fully functional health literacy level is not a matter of course.

Nutbeam also emphasises that functional health literacy levels are not just about having basic reading and writing skills, as even educated people may find it difficult to acquire, understand and use knowledge about health-related matters. People with limited reading skills will be exposed to less health-related information because of their reading difficulties and, thus, be restricted in their ability to act on the information they receive [27].

##### Interactive health literacy

Interactive health literacy is defined as having more advanced cognitive skills that may be used actively to extract information and form an opinion based on various types of communication [26].

Here, people will have the ability to handle large amounts of information from many different sources, and the type of information is more extensive than with functional health literacy. A person with an interactive health literacy level will be able to relate to information from different sources (which may occasionally contain contradictory information) and, on this basis, make an informed choice. In addition, such persons will have the ability to adjust knowledge from one area to another situation or new circumstances. People with high interactive health literacy levels are active in their contact with the health care system [26, 27].

##### Critical health literacy

Critical health literacy means more advanced cognitive skills that – combined with social skills – may be used to critically analyse information [26, 27]. The ability to relate critically to information increases the ability to make informed choices, allowing people to take control of the factors that affect their own health. Having critical health literacy allows persons to take ownership of their own life situation, thereby creating empowerment. Here, empowerment is seen as a process in which people are in control of decisions and have the ability to act on their wishes and their own lives.

Having the ability to acquire and relate critically to health information, citizens with critical health literacy receive advanced knowledge of health and the health care system. In addition, citizens with critical health literacy are often very proactive; that is, they are anticipatory and make an effort to prevent diseases. At the same time, they are outgoing and make demands [26].

##### Health literacy and social groups

A major international study (which Denmark was not part of) showed that certain population groups across countries have particularly restricted health literacy. In the Netherlands, 47% of the participants had limited health literacy levels. These were especially population groups who, in their own estimation, suffered from a poor health, had no or inferior education, were older than 75 years and had a low socioeconomic status. The Netherlands was the country in which the study found the highest health literacy levels on average. Therefore, it is remarkable that 47% of the Dutch population have what the study calls limited health literacy [26, 29].

Several other studies also show clear social inequality with respect to health literacy, as in some social groups, there are many more who find it difficult to communicate with the HPs about their problems or to understand the necessary information [3]. A connection is also seen between low health literacy and a perception of own health as poor, inappropriate health behaviour, risk of disease development and chronic disease management [30].

In society, health literacy is, for instance, expressed as the probability of not having a mammogram. Here, the risk of an unskilled woman not having a mammogram is about 25% higher than what applies for white-collar workers at lower- and middle-class levels. At the same time, the probability of not being examined is much higher among women in management positions and self-employed women. Thus, here, there is a more inverse U-shaped relationship when it is measured in relation to the women’s own social positions [11].

Several other studies support these results [3, 30–32]. The reason for this correlation is thought to be the health literacy level. The unskilled worker in a low social position does not have the examination because she does not have the ability or the surplus energy to understand and respond to the health information concerning the examination. On the other hand, the woman in a managerial and high social position has critical health literacy and can make an active choice not to be examined. It is essential to understand that even though the result is the same – that you do not have a mammogram – the underlying causes are completely different.

Health literacy level may be seen as an explanatory factor for a different starting point in an individual’s encounter with a HP [3, 11, 33]. Health literacy is thus expected to be correlated with trust and the game theory will consider precisely this in the encounter between patients at different health literacy levels and the HPs.

#### Game theory

Game theory is about strategic situations that can be analysed as a game. The basic idea behind game theory is that a conflict situation can be described based on the conflicting parties (players), their actions (strategies) and the outcome (benefit) arising from the actions of the parties [34]. One of the most well-known illustrations of the theory is called the prisoner’s dilemma [35]. Game theory has been used in many different connections to try to explain possible conflicts. It has also been used to explain matters in relation to patients and the health care system [36–38].

The following section will – by means of the game theory – illustrate what may happen in the encounter between patient and HP depending on the patient’s health literacy. In the established scenarios, the deciding factor is the patient’s expected ability to complain, as the risk of the patient complaining may change the HP’s behaviour [36, 39].

In the introduction, we established a positive correlation between social group and level of health literacy. Moreover, we also identified the positive correlation between willingness to cooperate and trust in the health care system. In the game-theoretic part, we derived the condition for a “u-shaped” relationship between social group and level of cooperation, depicted in the lower-left quadrant. Taken together, we obtain the main result of a “u-shaped” relationship between level of health literacy and trust in the health care system.

The expected ability to complain is to a large extent linked to health literacy and the ability to make demands in connection with the treatment. This ability may therefore explain why not everyone is necessarily receiving the same treatment within the health care system and do not have the same degree of trust [39]. Following the established scenarios, theoretical expectations are outlined with respect to the degree of trust at three different health literacy levels. In a simultaneous one-shot and non-cooperative setting, defection is a strictly dominating strategy choice for both parties, see Table 1.

**Table 1:** The perceived encounter between a health professional and patients (seen from the perspective of the patients)

	Patient	
	C	D
Health professional		
C	3/3	−2/4
D	4/−2	0/0

Numbers indicate benefits

However, the interaction between the HPs and the patients does not exactly match the original circumstances of the prisoners’ dilemma game. Assume that the HPs do not engage in a prisoner’ dilemma game but will always choose C. However, because of general lack of trust to institutions, people might think (perceive) that they do. In Table 1, the payoffs are therefore seen from the perspective of the patients, given their perception about how the HP will react.

The aim now is to set up a behavioural model based on the situation described in Table 1 that can explain the occurrence of a “inversely u-shaped” relationship between the probability of cooperation and the health literacy level ordered from lowest to highest. We will now argue that patients with low health literacy will perceive that the HPs are less likely to cooperate with the patients. We base this on the following observations.

It may take longer to explain specific correlations because patients with low health literacy levels have just a very basic knowledge of health. Possibly, several matters that are taken for granted in relation to other social groups will have to be explained. In a busy schedule, it may be hard to find time to pay sufficient attention to the socially disadvantaged, or tariffs may not allow more time or more money for the treatment of such patients [40]. It is therefore very likely that a HP with a busy schedule may cause patients with low health literacy to defect.

This may foster a perceived lack of trust in the willingness of the HPs to cooperate with patients with low health literacy. We should also describe more precisely what is implied by the strategy of defection. What behaviour will count as HPs “not cooperating” for patients from a low social group? They may have to deal with a great deal of information, and their (assumed) functional health literacy level does not enable them to relate to large amounts of information. They may, therefore, have difficulties cooperating because they do not always have the ability to translate and understand what they need to do to cooperate with the HP. At the same time, the patient’s ability to complain and make demands with respect to treatment is poor, and the HPs are therefore not pressured into improving their efforts. This again reinforces the lack of trust among patients in low social groups that the HPs will not cooperate.

A defecting patient may be described in terms of not complying with the HPs’ directions (called compliance) or having difficulty expressing symptoms and experiences in ways so that the HP is able to make a correct diagnosis [41]. In our model, we, first, simply assume that the higher the social group, the more trust these patients have in the HPs.

The second effect to be included is the perceived benefit to the patient from interacting with the HPs. Here, a patient with a high level of health literacy is associated with a higher risk of complaint and the determination to seek another physician or take action if not satisfied. In addition, such patients may already have examined their own health problem from home and have a clear attitude to what treatment they want.

In our interpretation, this will result in the patient with high health literacy perceiving the benefit from the interaction with the HPs as being lower than patients with lower health literacy would, in particular, if we include opportunity costs of being able to seek treatment elsewise. Thus, we suggest that the more health literacy a patient holds, the less perceived benefit from the interaction with the HP.

We are now in the position to formally model these considerations into one game-theoretic model based on the situation described in Table 1 (for a full description of the model setup and the simulation, see the appendix). Assume the existence of 10 social groups, denoted *g* = 1, 2, ⋯, 10. These social groups differ in two respects as measured by the average behavior for a group member. First, the perceived probability that the *HPs* will cooperate. Second, their perceived value of cooperation.

**Fig. 1:**
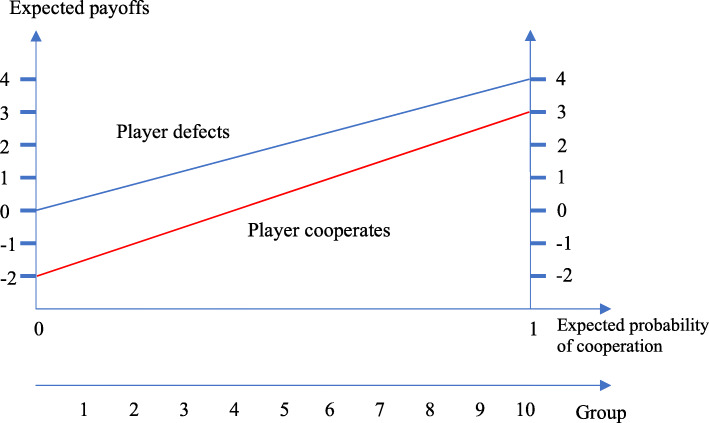
Expected payoffs from cooperation and defection depending on the expected probability that health personal will cooperate*.* Note: ${P}_g^C=0.1g$. For calculations of expected payoffs, see appendix

In Fig. 1, we show how the expected payoffs for the patients from cooperation and defection relate to the expected probability that health personnel will cooperate, ${P}_g^C$, based on the numbers in Table 1. The larger ${P}_g^C$, the larger the expected payoff from playing any of the two strategies. Note however, that defection always yields higher expected payoff than cooperation, no matter the size of ${P}_g^C$. Consequently, no cooperation can be expected. We need to invoke several behavioral assumptions that support cooperation rather than defection.

Our first hypothesis is that ${P}_g^C$ is positively related to *g*; belonging to a group with high health literacy increases the tendency to trust that the HPs will cooperate. In Fig. 1, this is illustrated by letting groups be uniformly distributed on the x-axis, where group 1 expects only 10% probability that HP will cooperate, while group 10 expects 100% cooperation.

The second behavioral hypothesis increase the likelihood of cooperation is to assert that people care not only about own welfare but also about the welfare of others who are affected by their decisions. More precisely, we assume that patients have “other-regarding” preferences in the sense that when evaluating the payoff of a strategy combination, they also derive utility from the wellbeing of their opponent. Based on the payoffs in Table 1, it turns out that if a player cares half as much about the opponent’s payoff than own payoff (both choosing cooperation), then the total payoff for the player is now 4.5 compared to the previous 3 when not caring at all.

According to Thaler [42], an important aspect of preferences that has received a great deal of attention from behavioral economic theorists is exactly these “other-regarding preferences”. Empirical findings have shown that in one-shot prisoners’ dilemma games, about 40 to 50% of subjects cooperate [43]. Similarly, people cooperate in public goods environments when the rational selfish strategy is to give nothing. In the words of Thaler ([42], 1593), “The easiest way to summarize this literature is to say that Humans are nicer and more mannerly than Econs. Specifically, their first instinct is to cooperate as long as they expect others to do likewise”.

**Fig. 2:**
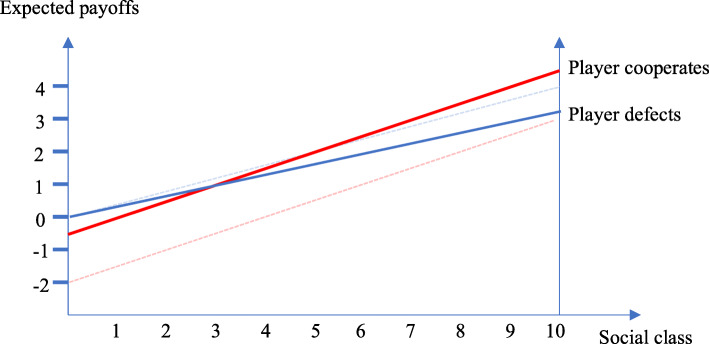
**a** Expected payoffs given the *perceived cooperation* effect. **b** Probability of cooperation *given the perceived cooperation effect.* Note: $\gamma =0.4,{P}_g^C=0,1\bullet g$. *γ* is the weight assigned to other players’ payoff compared to own payoff. The individual level of ${P}_{ig}^C$ for patients belonging to group *g* follows the pert distribution: ${P}_{ig}^C\sim pert\left(0;0.1g;1\right)$

If we pair hypothesis 1 (that ${P}_g^C$ is positively related to *g*) with hypothesis 2 (including other-regarding preferences), we get what may be called *the perceived cooperation* effect. The expected benefit from cooperation is now shifted upwards, while the expected payoff from defection is titled downwards. In Fig. 2a, the dotted lines are the original payoff lines. For group 3 and upwards, the expected payoff from cooperation is now larger than from defecting.

In order to make a realistic model simulation and derive probability statements, we will assert that although groups on average follow the described patterns, randomization implies that for any group, there is a distribution of *g* with group average as the medium value. It is assumed that there is a probability that a member of a particular group exhibits behavior that belong to the average behavior of other groups, but the further away the other group, the smaller this probability (see appendix for a full description).

This result is portrayed in Fig. 2b: The perceived cooperation effect suggests that higher social groups have a higher trust in institutions therefore perceiving with a larger probability that the HPs will cooperate. When this is put into the frame where people care also about the opponent’s utility, then the larger the social group, the larger the probability of choosing cooperation.

Our third and final hypothesis is that belonging to a higher social group reduces the perceived value from cooperation. We call this effect *the perceived benefit effect*. This effect is included in the model by assuming that the more critical an individual is about the expected treatment, the lower the perceived value will be of that treatment. This translates into the value of mutual cooperation between the patient and the HPs by assuming that the higher the group number, the lower the perceived value from cooperation.

**Fig. 3:**
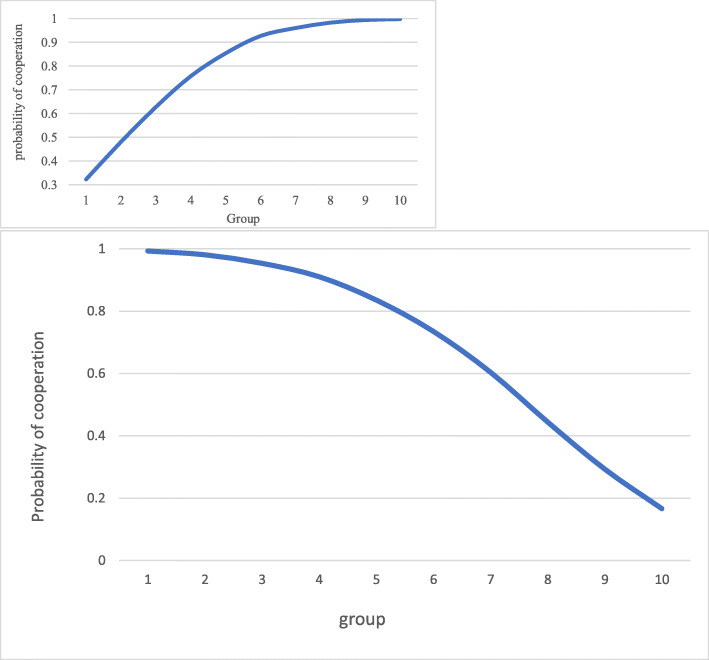
**a** Expected payoffs given the *perceived benefit* effect. **b** Probability of cooperation given the *perceived benefit* effect. Note: *γ* = 0.4, *C*_*g*_ = 3 − *α* ∙ (*g* − 1), where *C*_*g*_ is the payoff from mutual cooperation for group *g*. The parameter *α* > 0 measures how much from the original value is subtracted when moving to the next group. *α* = 0.1 is used in the model

Figure 3a shows, in contrast to Fig. 2a, that the expected payoff from cooperation is falling below the expected benefit from defection for higher social groups. For lower groups, cooperation does now on average yield more expected payoff than defection due to the inclusion of the other players payoff into own expected payoffs. For larger *g*, the reduction in payoff from cooperation dominates that effect thus giving these groups lower expected payoffs from cooperation than from defection. This is reflected in Fig. 3b: The larger the social group, the smaller the probability of choosing cooperation.

**Fig. 4:**
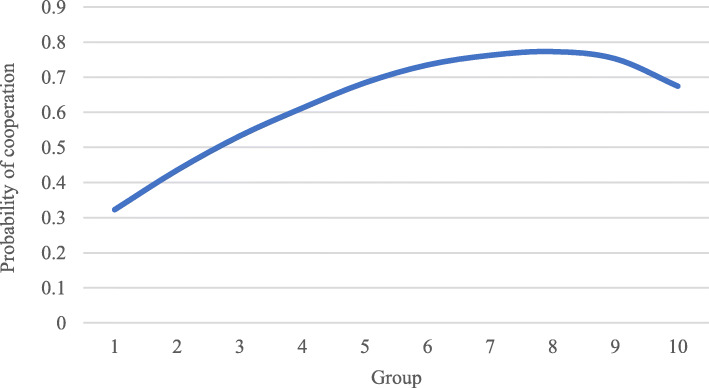
The combined cooperation effect and benefit effect

In summary, the *perceived cooperation effect* suggests that higher social groups have a higher trust in institutions and, therefore, perceive with a larger probability that the HPs will cooperate. The result is that the larger the social group, the larger the probability of choosing cooperation. In contrast, *the perceived benefit effect* gives the opposite result. Here, higher social groups assign a lower value to the benefit from cooperation. Consequently, they are less likely to cooperate. Having two countervailing effects, we can now estimate the combined effect. In Fig. 4 below, we show the result of simulating the combined cooperation and benefit effect given the parameter values used; the middle social groups exhibit the largest probability of cooperation.

The two parameters α and γ determine the shape of the curve. γ determines the speed of the willingness to cooperate. The more people care about other people’s utility, the more they are willing to cooperate. α, on the other hand, reduces the probability of cooperating. For a wide range of relative and absolute values of these two parameters, we find the inverse u-shaped relationship as depicted in Fig. 4 above.

**Fig. 5:**
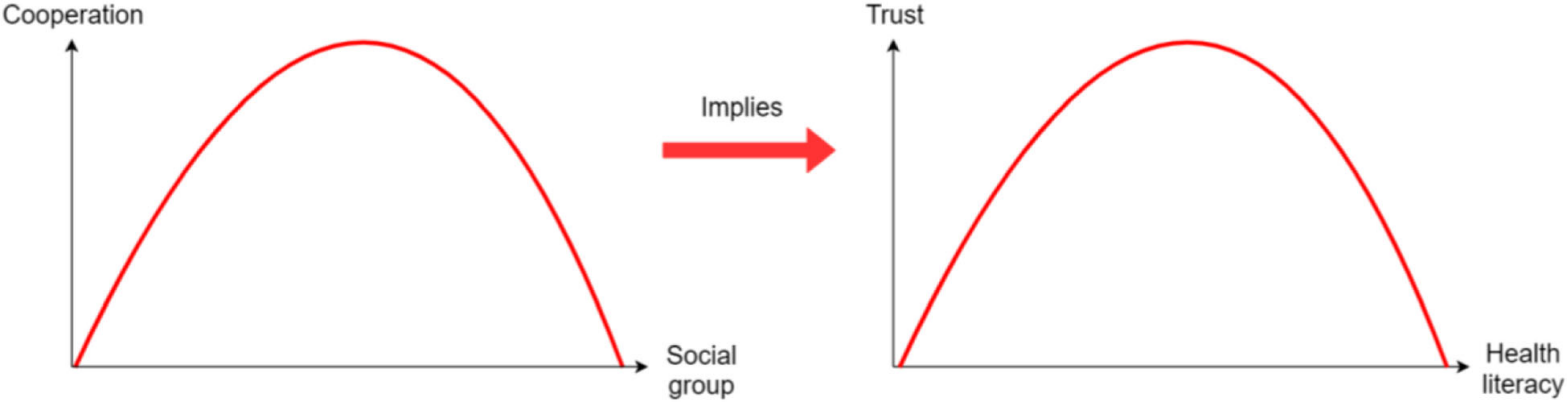
The inverse u-shaped relationship between health literacy and trust

We have derived the condition for a “u-shaped” relationship between social group and level of cooperation. Given the positive correlation between social group and level of health literacy and the positive correlation between willingness to cooperate and trust in the health care system, this also implies an inverse u-shaped relationship between levels of health literacy and trust, as shown in Fig. 5.

In general, the game theory thus shows possible correlations and options in the encounter between HP and patient. It illustrates how the outcome of the encounter between HP and patient may depend on the patient’s social group and the degree of trust that is associated with the encounter.

## Materials and methods

### Setting

The study was carried out in Denmark. The Danish healthcare system is universal and based on the principles of free and equal access to healthcare for all citizens. The healthcare system offers high-quality services, the majority of which are financed by general taxes [44].

### Definition of social groups

This study is based on Lars Olsen’s definition of the characteristics of various social groups [45]. In this section, Olsen’s definition is presented along with some proposals for relevant adaptations of the definition as the starting point for the study.

Olsen divides the society into five social groups, and the classification is based on three dimensions: level of education, income level and affiliation to the labour market. In today’s society, education is an important factor in determining a person’s position in society, whereas formerly, it was family name and income. Therefore, Olsen’s classification is primarily created based on education and affiliation to the labour market [45]. Olsen’s definition of social groups is intended for the age group 18–59 years, where students are excluded. This study wishes to examine the average degree of trust in the health care system among the population in different social groups. Thus, in the study, we have opted to *not* exclude students and people above 59 years (primarily retirees) because they are part of the average population. These choices and the placement of the two groups are elaborated in the next two sections.

### Operationalisation of social groups

The classification is based on three variables that contain information about the following:

1. Highest completed education
2. Main occupation
3. Personal annual income before tax.

Respondents who have not answered the question about income are not classified into a social group, as it is not known if they earn more than twice the typical income and, thus, should be classified in social group 1 or 2.

#### Social group 1 (upper group)

This social group is characterised by citizens who have an income of more than three times the typical income. The typical income is set at the median of income in 2015, that is, DKK 230,140 [46]. In the data, the income is divided into various intervals. The limit in the upper group is set at DKK 700,000 because this is best in line with three times the typical income.

The classification for this group is reached as follows:

1. Having a personal annual income of more than DKK 700,000, corresponding to more than DKK 58,334 per month.

#### Social group 2 (higher middle group)

This social group is characterised by persons having an academic education and persons who have an income of between two to three times the typical income.

The classification for this group is reached as follows:

1. Having a personal annual income of between DKK 500,000 and 699,999
2. Having a main occupation as senior white-collar worker (academic education or
3. management responsibility) and not being placed in social group 1
4. Having a long higher education (5 years or more) as the highest completed education and not being placed in social group 1.

#### Social group 3 (middle group)

This social group is characterised by persons with short or medium-cycle higher educations and self-employed who do not earn more than twice the typical income.

The classification for this group is reached as follows:

1. Being self-employed as a main occupation and not earning more than two times the typical income
2. Having a short higher education (under 3 years) or medium-cycle higher education (3 to 4 years) as the longest completed education and not earning more than twice the typical income.

#### Social group 4 (working group)

This social group is characterised by persons with a vocational education, unskilled persons or persons not included in other social groups.

The classification for this group is reached as follows:

1. Having primary/secondary school education, general upper secondary education (high school/higher preparatory examination (HF)), vocational upper secondary education (HH/HTX/HHX), social and health care training (SOSU) or vocational education (crafts, trade, office, etc.) as the highest education and not being included in any of the other social groups.
2. Having unskilled work, specialised work, skilled work or lower white-collar work as a main occupation and not being placed in any of the other social groups.

#### Social group 5 (lower group)

This social group is characterised by persons who are outside the labour market for 4/5 of the year. As mentioned earlier, there is no variable indicating how long people are away from the labour market. Therefore, everyone outside the labour market is classified into this group. People who are classified into this group cannot be included in any of the other groups, regardless of the level of education or income. The classification for this group is reached by including people whose main occupation is as homemakers, unemployed receiving unemployment benefits, unemployed without any benefits or early retirees.

### Data and statistical method

The study uses secondary data from the a survey from Trygfonden and Monday Morning [10]. The questionnaire asks about trust, using the following questions:

*Imagine that you will need the following sectors of the health care system. To which degree do you believe that you will have a satisfactory process*

*1 with a medical practitioner?*

*2 with a medical specialist?*

*7 in psychiatric?*

*8 at a public hospital (non-psychiatric)?*

*9 at a private hospital (non-psychiatric)?*

All five questions had the following response options: “Not at all”, “to a lesser extent”, “to some extent”, “to a large extent”, “to a very large extent” and “do not know”.

The data have been categorised in such a way that the response options “to a large extent” and “to a very large extent” express trust, while the responses “not at all”, “to a lesser extent” and “to some extent” express a lack of trust. “Do not know” answers were left out of the survey. The statistical method used is binary regression analysis.

## Results

### Presentation of social groups

The characteristics of the various social groups are shown in Table 2. The table shows that the largest group is social group 4, which accounts for 41.9%, while the smallest social group is social group 1. This is as expected and in accordance with Olsen’s expectation of the distribution. However, social groups 1 and 2 seem to be larger and social group 5 smaller than expected by Olsen. He expected that social groups 1 and 2 would constitute 1 and 9%, respectively, while he expected that social group 5 would account for approximately 20% [45]. It is not possible to say whether the difference is due to the adjustments of the classification or whether it represents an imbalance in the representativeness of the data in relation to social groups.

**Table 2:** Characteristics of the social classes in the dataset

	Characteristics of the social classes				
	Social class 1	Social class 2	Social class 3	Social class 4	Social class 5
Number	246	1133	1411	2449	606
Number in %	4.21%	19.38%	24.14%	41.9%	10.37%
Average age	49.4 years	46.5 years	49.3 years	48.2 years	46.5 years
Women in %	25.2%	40.1%	57.1%	53.4%	64%
Chronic disease in %	20.7%	20.3%	26.6%	31.7%	59.6%
Errors in %	40.7%	35%	34.3%	29.7%	44.1%

As shown in the table, the average age is roughly the same in the various groups, but there is a clear difference with respect to the share of women and share of people who have a chronic disease. In both these cases, there is an increase from social group 1 to social group 5. Finally, the table also shows that there are more people in social groups 1 and 5 who have previously experienced errors within the health care system. The presented characteristics may emphasise a possible need to adjust for these variables, but they may also clarify the differences that are inherently present in the various social groups.

### Trust in public hospitals in relation to social group

In analysing the average trust of the respondents in the various groups, the probability of trust in the public hospital is as shown in Table 3. The probability is calculated based on the weighted analysis (Model 1).

**Table 3:** Probability of trust in public hospitals calculated based on Model 1

Public hospital: Probability of trust	
Social class 1	48.3% (38.0%; 58.6%)
Social class 2	51.2% (48.2%; 54.3%)
Social class 3	51.9% (44.8%; 59.1%)
Social class 4	55.7% (48.9%; 62.4%)
Social class 5	48.1% (39.8%; 56.5%)

It is apparent from Table 3 that social group 5 (the lowest social group) has the least trust in the public hospital at an estimated 48.1% (39.8, 56.5%). Social group 1 has the second-lowest trust in the public hospital at an estimated 48.3% (38.0, 58.6%), and social group 4 has the highest trust at an estimated 55.7% (48.9, 62.4%).

Table 4 below shows the probability of trust in the public hospital in the various social groups through the three different analyses. Social group 2 is the reference group, and the table has been calculated in risk differential. Using Model 1, it is apparent that the social group 4 estimate is 4.5% points higher than in social group 2. This is significant because the safety interval does not contain zero, as shown by an asterisk.

**Table 4:** Probability of trust in the public hospital, calculated in risk differential

Public hospital: Probability of trust			
	Model 1: Weighted	Model 2*	Model 3**
Social class 1	−2.9% (− 10.2%; 4.4%)	− 4.2% (− 11.4%; 3.0%)	− 3.8% (− 10.9%; 3.3%)
Social class 2	0 (reference)	0 (reference)	0 (reference)
Social class 3	0.7% (− 3.4%; 4.8%)	0.4% (− 3.7%; 4.5%)	−0.3% (−4.4%; 3.8%)
Social class 4	4.5% (0.8%; 8.2%) *	3.5% (−0.2%; 7.3%)	2.7% (− 1.1%; 6.5%)
Social class 5	−3.1% (− 8.4%; 2.2%)	− 3.2% (− 8.5%; 2.1%)	−1.7% (− 7.5%; 4.1%)

* Model 2 is adjusted for gender, age and city/town size

** Model 3 is adjusted for gender, age, city/town size, errors, chronic disease, self-assessed health and private/public employee

Model 2 shows the estimates of trust in the public hospital when adjusted for gender, age and city/town size. The difference between social groups grows in social groups 1 and 5 and decreases in social groups 3 and 4. Model 2 shows no statistically significant difference in the trust in the public hospital between social group 4 and social group 2.

Model 3 shows the estimates of trust in the public hospital when – in addition to the adjustments in Model 2 – adjustments are also made for previously experienced errors, chronic diseases, self-assessed health and private/public employee. Table 4 shows that the difference between the various social groups in Model 3 declines for everyone. It is noteworthy that social group 3 changes from a positive estimate, that is, higher trust than social group 2, to a negative estimate in Model 3. There is a striking change in the social group 5 estimate from Model 2 to Model 3, from − 3.2% points to − 1.7% points.

In summary, there is – as expected – a low level of trust in social group 5. As the level of health literacy increases, the trust also increases to social group 4. Then the trust decreases, and at social group 1, it is at approximately the same level as at social group 5.

## Discussion

Previous studies have established a clear positive association between social groups and level of health literacy [26, 29] and showed that higher levels of health literacy led to higher levels of trust in physicians and in the health care system [20–22]. In this study, both the theoretical model and the empirical data showed that the relationship was better described as an inversely U-shaped curve and the reason for this correlation was the health literacy level. The correlation pointed to an association where functional (low) and critical (high) level of health literacy was associated with low trust, and interactive (medium) health literacy was associated with high trust. Thus, the results confirmed the presumption that health literacy level can be seen as an explanatory factor for different starting points in individual’s encounter with the health care system [3, 11, 33]. The findings are in line with the results of Diderichsen et al who found that both low and high level of health literacy can lead to women’s choice of not having a mammogram [11].

The theoretical model was built on an assumption that HPs always will corporate but that the patients with low and high level of health literacy does not always experience this. The reason for this might be, that in a busy schedule, it can be hard to find time to pay sufficient attention to the socially disadvantaged with low health literacy, or tariffs may not allow more time or more money for the treatment of such patients. On the other hand, patients with high level of health literacy might have higher demands for their treatment as they are well-informed and able to seek second opinions. Therefore, the HPs might not be able to comply with the wishes and expectations of patients with high level of health literacy. Several studies showed that trust in the health care system is crucial for most patients [2] and the foundation for a good and well-functioning health care system [3, 4]. In today’s health care system with scarce resources, it is of urgent importance to better understand the consequences of different health literacy levels among patient groups. This can potentially strengthen the quality of treatment, the patient satisfaction and the use of available resources and at the same time help to decrease inequality in health across social groups. The study was carried out in Denmark where the health care system is universal and has free and equal access to healthcare for all citizens. Nevertheless, the findings might inform other types of health care systems to understand the need for tailored approaches to patients’ level of health literacy and their social status.

In this study, the distribution of participants across social groups differed slightly according to what was expected [45]. Social groups 1 and 2 was larger and social group 5 smaller than expected. It is not possible to say whether the difference is due to the adjustments made to the classification or whether it represents an imbalance in the representativeness of the data. Furthermore, there were differences with respect to the share of women and share of people who have a chronic disease between the social groups and more people in social groups 1 and 5 had previously experienced errors within the health care system. In Model 2 and 3 we adjusted for gender. The remaining characteristics may emphasise a possibility for further adjustments, but they may also clarify the differences that are inherently present in the various social groups. Finally, the.

model is general and adjusting it for other factors could be useful in future research such as severity of the disease or condition, time living with the disease or condition, urgency of treatment, type of provider in case you have more than one public health provider, etc.

## Conclusion

The purpose of this paper was to investigate whether higher health literacy leads to higher trust in public hospitals. Health literacy and game theory combined formed the basis for the theoretical approach. Health literacy could be divided into three different levels: functional, interactive and critical health literacy. Several studies supported the fact that there was a strong positive correlation between social groups and health literacy. It was therefore assumed that the highest social group had a *critical* health literacy level, the middle group an *interactive* and the lowest group a *functional* health literacy level. Furthermore, previous studies have shown that higher levels of health literacy led to higher levels of trust in the health care system. We challenged this result and wanted to see if a high level of health literacy could potentially lead to a lower level of trust.

In the game-theoretic model, we analysed the effect of health literacy on the perceived cooperation of the health care system and the perceived benefit from the health care system and how these effects spill over into the trust in the health care system – with focus on how to explain a systematic variation in trust among the social groups. The theoretical model was built on the interaction of patients and health personal similar to the original prisoners’ dilemma game. The interaction, however, was changed slightly to cover this interaction.

Patients in lower socials groups have lower institutional trust and may also have had experienced what might have seemed as non-cooperative behaviour. By coupling this with the health literacy effect, the model showed under which circumstances we find an inversely u-shaped relationship between social group and trust in the health care system.

The encounters between a patient and a HP varied depending on the patient’s social group. The people in the highest social group got something out of being critical, as they were more capable of convincing the HP to give them the treatment they wanted. This demand strategy was possible due to a credible potential threat of using resources to complain and seek other physicians for a second opinion.

People from higher social groups benefited from being critical and displayed a lower level of trust. With respect to the middle group, game theory could explain that the physician’s assessment to a large extent depended on the physician’s professional judgment, giving people in the middle group good reason to show trust. Conversely, the physician did not have as much incentive to treat the low social group because it was more time consuming and the socially disadvantaged patient did not always have the skills to cooperate with the physician.

The combination of the perceived cooperation effect (higher social groups have a higher trust in institutions) and the perceived benefit effect (higher social groups assign a lower value to the benefit from cooperation). Having two countervailing effects,

we can now estimate the combined effect. In Fig. 4 below, we show the result of simulating the combined cooperation and benefit effect given the parameter values used; the middle social groups exhibit the largest probability of cooperation.

Such inverse u-shaped relationship between levels of health literacy in social groups and trust in the health care system implies that the middle social group would have the highest degree of trust, while the higher and lower social groups would have less trust.

These theoretical expectations were matched by the empirical findings. Overall, the empirical pattern based on quantitative, binary regression analyses of data from a study of the Danes’ relationship to the overall health care system showed that both social groups 3 and 4 had the highest degree of trust (the middle group), while social groups 1 and 5 had a lower degree of trust. This might be interpreted in the way that functional and critical health literacy are associated with a lower level of trust, while interactive health literacy is associated with higher level of trust in the public health care system.

In perspective, the needs of each patient and the conditions for the encounter with a HP should be taken into account when the function of the health care system is optimised. It is important that HPs understand that some patient groups have a higher chance of cooperation or defection than others. Screening the patients in this way can save resources and increase trust. Future research could test in more detail the theoretical explanations of the study with respect to variations in the degree of trust. This could be done through qualitative interviews that provide more in-depth knowledge and an inside perspective from the patients in all social groups. Another interesting study could be an investigation into the causes of potential trust variations among different health care sectors.

## Data Availability

The datasets analysed during the current study available are from the corresponding author on reasonable request.

## Appendix

Here, we present the full description of the model and the simulation of the analysis presented in section 2.

The general notation: Let ${\pi}_{ig}\left({s}_i,{P}_g^C\right)$ be the utility to player *i* belonging to group *g* from playing *s*_*i*_, when its opponent with perceived probability ${P}_g^C$ plays cooperation Let ${p}_{ig}\left({s}_i,{P}_g^C\right)$ be the direct payoff for player *i*: ${\pi}_{ig}\left({s}_i,{P}_g^C\right)={p}_{ig}\left({s}_i,C\right)\bullet {P}_g^C+{p}_{ig}\left({s}_i,D\right)\bullet \left(1-{P}_g^C\right)$. By using the numbers presented in Table 1, we can derive general expressions for the expected utility of playing cooperation and defection:

$$ {\pi}_{ig}\left(C,{P}_g^C\right)={p}_{ig}\left({C}_i,C\right)\bullet {P}_g^C+{p}_{ig}\left(C,D\right)\bullet \left(1-{P}_g^C\right)=3{P}_g^C-2\bullet \left(1-{P}_g^C\right)=5{P}_g^C-2\kern1.25em (1a) $$

$$ {\pi}_{ig}\left(D,{P}_g^C\right)={p}_{ig}\left(D,C\right)\bullet {P}_g^C+{p}_{ig}\left(D,D\right)\bullet \left(1-{P}_g^C\right)=4{P}_g^C-0\bullet \left(1-{P}_g^C\right)=4{P}_g^C\kern3.25em (1b) $$

The model consists of four additional elements:

**(1) Other regarding preferences.**

Other regarding preferences are defined as follows: for two players, *i* and *j*, and the strategy profile (*s*_*i*_; *s*_*j*_), the utility for player *i* is given by:

$$ {\pi}_i\left({s}_i;{s}_j\right)={p}_i\left({s}_i;{s}_j\right)+{\gamma}_i\cdotp {p}_j\left({s}_i;{s}_j\right),\kern0.5em {\gamma}_i\ge 0\kern1.75em (1c) $$

*γ*_*i*_ measuring the relative weight assigned to the opponent’s payoff.

**(2) Relationship between** ${\boldsymbol{P}}_{\boldsymbol{g}}^{\boldsymbol{C}}$ **and** ***g*** **– and (1) is called the perceived cooperation effect.**

We let:

$$ {P}_g^C=0.1\cdotp g\kern1.25em (1d) $$

**(3) Relationship between** ***C*** **and** ***g*** **– and (1) is called the perceived benefit effect.**

We let:

$$ {C}_g={C}^o-\alpha \bullet \left(g-1\right),\alpha >0\kern1.5em (1e) $$

Here *C*^*o*^ is the payoff from mutual cooperation in Table 1, and *C*_*g*_ is the payoff from mutual cooperation for group *g*.

**(4) The randomization process and the simulation.**

The simulations are made in excel, using the simulation tool of Modelrisk by Vose software. The simulation is as follows. First, the parameter values for *α* and *γ* are set. For each group, *g*, and for each run, *k*, of the model, a value of ${P}_{ig}^C={P}_k^C$ is randomly drawn from the distribution *pert*(0; 0.1*g*; 1).

**Part 1: Deriving the** ***perceived cooperation*** **effect.**

This is done by adding (1), (2) and (4) to (1a) and (1b).

Including (1c) and into (1a) and (1b) yield:

$$ {\pi}_{ig}\left(C,{P}_g^C\right)=\left[5-{\gamma}_i\right]\bullet {P}_g^C+{\gamma}_i\cdotp 4-2 $$

$$ {\pi}_{ig}\left(D,{P}_g^C\right)=\left[4-2{\gamma}_i\right]\bullet {P}_g^C $$

Using *γ*_*i*_ = 0.4:

$$ {\pi}_{ig}\left(C,{P}_g^C\right)-{\pi}_{ig}\left(D,{P}_g^C\right)=4.6\bullet {P}_g^C-0.4-3.2\cdotp {P}_g^C=1.4\cdotp {P}_g^C-0.4 $$

$$ {\pi}_{ig}\left(C,{P}_g^C\right)-{\pi}_{ig}\left(D,{P}_g^C\right)\ge 0=>1.4\cdotp {P}_g^C\ge 0.4=>{P}_g^C\ge 0.29 $$

Inserting (1d):

$$ {\pi}_{ig}\left(C,0.1g\right)=4.6\bullet 0,1g-0.4=0.46g-0,4 $$

$$ {\pi}_{ig}\left(D,0.1g\right)=3.2\cdotp 0,1g=0.34g $$

This is shown in Fig. 2a.

Further calculations yield:

$$ {\pi}_{ig}\left(C,{P}_g^C\right)-{\pi}_{ig}\left(D,{P}_g^C\right)=>{P}_g^C\ge 0.29=>0,1\bullet g\ge 0.29=>g\ge 2.9 $$

Without any variability, all groups larger than two will cooperate.

The randomization process: In the simulation, if ${\pi}_{ig}\left({C}_g,{P}_k^C\right)\ge {\pi}_{ig}\left(D,{P}_k^C\right)$, where ${P}_k^C\sim pert\left(0;0,1g;1\right),$ then this run is assigned a number of 1 and, otherwise, a 0. The simulation makes 10,000 runs of the model, and the probability of cooperation is calculated as the number of runs with a 1 divided by 10,000 (which is equal to the simulated mean). For each simulation (run) of the model, for each group, ModelRisk draws one number according to the *pert*(0; 0.1*g*; 1) distribution, such that the probability of drawing any number between 0 and 1 is determined by the pert function for that group. The simulation is shown in Fig. 2b.

**Part 2: Deriving the** ***perceived benefit*** **effect.**

The perceived benefit effect is found by adding (1), (3) and (4) to (1a) and (1b). This effect is then isolated by letting ${P}_g^C$ not be dependent on *g*. Also, *α* = 0.1, *C*^*o*^ = 3 and *γ*_*i*_ = 0.4.

Inserting (1c) and (1e) into (1a) and (1b) yield:

$$ {\pi}_{ig}\left(C,{P}_g^C\right)=\left(4.6-0,1\bullet \left(g-1\right)\right)\bullet {P}_g^C-0.4 $$

$$ {\pi}_{ig}\left(D,{P}_g^C\right)=\left[4-2{\boldsymbol{\gamma}}_{\boldsymbol{i}}\right]\bullet {P}_g^C=3.2\cdotp {P}_g^C $$

By setting ${P}_g^C=0.5$ for all *g*, we derive Fig. 3a.

Using the randomized model ${P}_{ig}^C\sim pert\left(0;0.5;1\right)$, we get a decrease in the probability of cooperating for an increase in *g*, and following the same simulation procedure as above, we derive the relationship between social group and probability of cooperation from isolating the perceived benefit effect, as depicted in Fig. 3b.

**Part 3: The combined model.**

For the combined model, we can simply use the model from part 2:

$$ {\pi}_{ig}\left(C,{P}_g^C\right)=\left(4.6-0,1\bullet \left(g-1\right)\right)\bullet {P}_g^C-0.4 $$

$$ {\pi}_{ig}\left(D,{P}_g^C\right)=\left[4-2{\boldsymbol{\gamma}}_{\boldsymbol{i}}\right]\bullet {P}_g^C=3.2\cdotp {P}_g^C $$

This yields the final result shown in Fig. 4.
